# Targeting GLI1 expression in human inflammatory breast cancer cells enhances apoptosis and attenuates migration

**DOI:** 10.1038/bjc.2011.133

**Published:** 2011-04-19

**Authors:** Z I Thomas, W Gibson, J Z Sexton, K M Aird, S M Ingram, A Aldrich, H K Lyerly, G R Devi, K P Williams

**Affiliations:** 1 Biomanufacturing Research Institute and Technology Enterprise (BRITE), Durham, North Carolina Central University, Durham, NC 27707, USA; 2 Department of Pharmaceutical Sciences, Durham, North Carolina Central University, Durham, NC 27707, USA; 3 Division of Surgical Sciences, Department of Surgery, Duke University Medical Center, Durham, NC 27710, USA; 4 Duke Comprehensive Cancer Center, Duke University Medical Center, Durham, NC 27710, USA

**Keywords:** IBC, GLI1, hedgehog, migration, XIAP, epidermal growth factor receptor

## Abstract

**Background:**

Inflammatory breast cancer (IBC) is an aggressive subtype of breast cancer with distinct molecular profiles. Gene expression profiling previously identified sonic hedgehog (SHH) as part of a gene signature that is differentially regulated in IBC patients.

**Methods:**

The effects of reducing GLI1 levels on protein expression, cell proliferation, apoptosis and migration were determined by immunoblots, MTT assay, Annexin-V/PI assay and conventional and automated cell migration assays.

**Results:**

Evaluation of a panel of breast cancer cell lines revealed elevated GLI1 expression, typically a marker for hedgehog-pathway activation, in a triple-negative, highly invasive IBC cell line, SUM149 and its isogenic-derived counterpart rSUM149 that has acquired resistance to ErbB1/2 targeting strategies. Downregulation of GLI1 expression in SUM149 and rSUM149 by small interfering RNA or a small molecule GLI1 inhibitor resulted in decreased proliferation and increased apoptosis. Further, GLI1 suppression in these cell lines significantly inhibited cell migration as assessed by a wound-healing assay compared with MCF-7, a non-invasive cell line with low GLI1 expression. A novel high-content migration assay allowed us to quantify multiple effects of GLI1 silencing including significant decreases in cell distance travelled and linearity of movement.

**Conclusion:**

Our data reveal a role for GLI1 in IBC cell proliferation, survival and migration, which supports the feasibility of targeting GLI1 as a novel therapeutic strategy for IBC patients.

Inflammatory breast cancer (IBC) is one of the most lethal and highly invasive forms of locally advanced breast cancer with a 5-year survival rate of <50%, which is significantly less than patients with non-IBC breast cancer (85%) (Hance *et al*, 2005; Cristofanilli *et al*, 2007). It is clear that IBC is phenotypically and molecularly distinct from other forms of locally advanced breast cancer and IBC tumours are particularly resistant to chemotherapeutic agents (Charafe-Jauffret *et al*, 2004; Lee *et al*, 2004; Cristofanilli *et al*, 2007). Gene expression profiling has linked genes such as LIBC (loss in IBC), Rho C, BP1 and E-cadherin to the IBC phenotype (Yamauchi *et al*, 2009). A gene expression profile that distinguished between IBC and non-IBC tumours identified sonic hedgehog (*SHH*) as being a part of a three-gene signature that was differentially regulated in IBC patients (Bieche *et al*, 2004). SHH, one of the three members of the mammalian Hh family of secreted ligands (Pathi *et al*, 2001), was noteworthy for being higher in patients who relapsed and had the most aggressive form of IBC (Bieche *et al*, 2004). Gene expression analysis of IBC and non-IBC tumour samples also identified Hh-pathway overexpression in IBC samples and that this was a predictor of the IBC phenotype (Van Laere *et al*, 2010).

Hh signalling in vertebrates is mediated by the downstream zinc-finger transcription factors of the Gli protein family (Kinzler *et al*, 1988), with GLI-mediated transcription being the final step in the Hh pathway. In the absence of Hh, the receptor patched (PTCH) inhibits Hh-pathway activity through the inhibition of smoothened (SMO), a GPCR-like receptor that transduces the signal for the phosphorylation and proteolysis of GLI3 into a repressor form. GLI3 translocates to the nucleus and represses Hh target genes. When present, Hh binds to PTCH, which releases its inhibition of SMO. SMO is then able to transduce the signalling cascade through the GLI1 transcriptional activator form, which then can regulate the expression of Hh target genes including *GLI1* and *PTCH*, making these genes markers for Hh-pathway activation (Ingham and McMahon, 2001; Kasper *et al*, 2006). Hh target genes are involved in cell proliferation, survival, differentiation, cell cycle, stem cells and invasion (Katoh and Katoh, 2009).

Dysregulation of the Hh-Gli pathway in cancer has been reported and reviewed extensively (Kasper *et al*, 2006; Scales and de Sauvage, 2008; Yang *et al*, 2009), with Hh-ligand-dependent autocrine and paracrine (Yauch *et al*, 2008; Theunissen and de Sauvage, 2009), and Hh-ligand-independent mechanisms of activation observed (Rubin and de Sauvage, 2006). Activation of GLI via gene amplification has been linked to tumourgenesis in a number of tumours, including ovarian (Chen *et al*, 2007), endometrial (Feng *et al*, 2007), prostate (Karhadkar *et al*, 2004) and oesophageal (Mori *et al*, 2006) cancers. GLI1 contributes to the invasiveness of pancreatic (Nagai *et al*, 2008; Inaguma *et al*, 2011), prostate (Karhadkar *et al*, 2004), melanoma (Das *et al*, 2009) and glioma (Wang *et al*, 2010a) cancers. GLI1 is overexpressed in the majority of breast cancers (Xu *et al*, 2010) and a role for GLI1 in breast cancer invasiveness has been reported (Kameda *et al*, 2009; Souzaki *et al*, 2011). GLI1 overexpression has clinical impact and in breast cancer correlates with poor patient prognosis (Ten Haaf *et al*, 2009). A number of approaches have been taken to block Hh-Gli signalling at different steps in the pathway including natural products (Hosoya *et al*, 2008), synthetic small molecule inhibitors such as cyclopamine derivatives (Williams *et al*, 2003), blocking monoclonal antibodies (Ericson *et al*, 1996) and small interfering RNA (siRNA) (Sanchez *et al*, 2004). More recently, there is mounting evidence for alternative mechanisms for the activation of Hh-Gli pathway components (Testaz *et al*, 2001; Riobo and Manning, 2007; Jenkins, 2009; Chinchilla *et al*, 2010; Teglund and Toftgård, 2010), with upregulation of GLI by non-canonical pathways independent of Hh/SMO signalling reported (Lauth and Toftgard, 2007; Fernandez-Zapico, 2008; Nolan-Stevaux *et al*, 2009; Rizvi *et al*, 2010).

In this study, we observed that the triple-negative, Basal-like IBC cell line SUM149, a widely used model for IBC (Ignatoski and Ethier, 1999; Hoffmeyer *et al*, 2005; Dong *et al*, 2007; Streicher *et al*, 2007; Aird *et al*, 2010; Wu *et al*, 2010; Singh *et al*, 2010a, 2010b), and rSUM149, an IBC cell line with acquired resistance to ErbB1/2 targeted agents (Aird *et al*, 2010), had significantly higher GLI1 expression than other cell lines tested and that targeting GLI1 by siRNA reduced cell proliferation and migration and increased apoptosis. We were able to quantify the effects of GLI1 silencing on migration using a high-content imaging assay that tracked multiple motility parameters of H2B-GFP-labelled cells. Our findings suggest that GLI1 has a role in the proliferation, migration and survival of the IBC cell lines.

## Materials and methods

### Cell culture and reagents

SUM149, SUM44 and SUM190 cell lines were obtained from Asterand (Detroit, MI, USA) and SK-BR-3, MCF-7, BT474 and MDA-MB-231 cell lines were from ATCC (Manassas, VA, USA). Human mammary epithelial (HMEC) cells were from Duke University Medical School. These cell lines were grown as described previously (Aird *et al*, 2008). rSUM149 and rSUM190 were grown as described (Aird *et al*, 2010). Sonic hedgehog (ShhN) recombinant protein was from R&D Systems (Minneapolis, MN, USA). The hybridoma expressing 5E1 monoclonal antibody developed by Thomas Jessell (Ericson *et al*, 1996) was obtained from the Developmental Studies Hybridoma Bank developed under the auspices of the NICHD and maintained by the University of Iowa, Department of Biology (Iowa City, IA, USA). The activity of the ShhN ligand and ability of 5E1 to block this activity was confirmed in the standard C3H10T1/2 differentiation assay for Hh activity (Pepinsky *et al*, 1998; Williams *et al*, 1999; Wang *et al*, 2000). The ShhN ligand had an EC_50_ value of 2 *μ*g ml^–1^ in this assay (Supplementary Figure 1A) comparable to previously reported values (Williams *et al*, 1999; Pathi *et al*, 2001) and 5E1 was effective at blocking this activity compared with control Ab (Supplementary Figure 1B).

3-Keto-*N*-(aminoethyl-aminocaproyl-dihydrocinnamoyl)-cyclopamine (KAAD-cyc), tomatidine and GANT58 were from Calbiochem (La Jolla, CA, USA) and dissolved in DMSO at 2 mM stock concentration.

### Real-time PCR

Total RNA was extracted from cells using an RNA extraction kit (Ambion, Austin, TX, USA). RNA (0.5–1 *μ*g) was converted into cDNA using the high-capacity cDNA RT–PCR kit (Applied Biosystems, Carlsbad, CA, USA). Quantitative real-time PCR (qRT–PCR) analyses were carried out in an ABI Prism Fast 7500 system (Applied Biosystems). Gene expression for *GLI1* (Hs00171790_m1), *SHH* (Hs00179843_ml), *E-cadherin* (Hs00170423_ml) and *Snail* (Hs00195591_ml) were analysed using the indicated pre-designed Taqman gene expression assays (Applied Biosystems). A *β*-actin primer set (Ambion) was used as an internal control. Folds difference (2^−ΔΔCT^) were calculated as described previously (Aird *et al*, 2008) and represent changes normalised to a reference HMEC cell line.

### Immunoblot analysis

Cells were lysed in 50 mM Tris–HCl, pH 8.0, 150 mM sodium chloride, 1.0% Igepal CA-630 (NP-40), 0.5% sodium deoxycholate and 0.1% sodium dodecyl sulphate supplemented with a protease inhibitor cocktail (Sigma, St Louis, MO, USA). Cell lysates were separated by SDS–PAGE and immunoblot analysis was carried out as previously described (Aird *et al*, 2008, 2010). Membranes were probed with a primary antibody against GLI1 (R&D Systems) at 1:10 000 dilution overnight at 4°C. Probing with other primary antibodies for X-linked inhibitor of apoptosis protein (XIAP) and procaspase 9, and appropriate HRP-conjugated secondary antibodies was carried out as previously described (Aird *et al*, 2008). Bands were visualised using SuperSignal West Dura Extended Duration Substrate (Pierce, Rockford, IL, USA). As a loading control, membranes were stripped and reprobed with a primary antibody against *β*-actin (1:50 000; Sigma).

### RNA interference

Silencer pre-designed siRNA specific for GLI1 (siRNA ID number 115642), Silencer pre-designed siRNA specific for monoamine oxidase (Maob) (siRNA ID number 49300) and Silencer Negative Control siRNA were from Ambion. An alternative GLI1 siRNA was Dharmacon siGenome SMART pool Human GLI1 (Cat. # M-003896; Thermo Scientific, Lafayette, CO, USA). Cells were transfected according to the manufacturer's instructions either with the GLI1-specific siRNA or with the control siRNAs at 100 nM using lipofectamine 2000 (Invitrogen, Carlsbad, CA, USA). Downregulation of GLI1 expression was measured 72 h after transfection by RT–PCR and immunoblot analysis. Lipofectamine alone was used as a control in all siRNA experiments. In some experiments, cells were viewed by phase light microscopy using an Olympus IX51 inverted microscope at a × 100 magnification and cells were visualised using the NIS-Elements BR (Nikon, Melville, NY, USA) imaging software. Pictures were taken with a Nikon Digital Sight Camera.

### Cell proliferation

Cell proliferation was measured as described previously (Aird *et al*, 2008). Cells were seeded at a density of 2000 cells per well in a 96-well plate and cultured for 72 h. Culture medium was aspirated and replaced with fresh media on the day of treatment. After treatment with agents, culture media was aspirated, and 5 mg ml^–1^ 3-[4,5-dimethylthiazol-2-yl]-2,5-diphenyl tetrazolium bromide (MTT; Sigma) was added. Cells were incubated at 37°C for 4 h to allow formation of the coloured formazan product. The reagent was aspirated and 100 *μ*l of DMSO was added in each well. Plates were incubated for a further 2 h and then absorbance (550 nm) was measured using a SpectraMax plate reader (Molecular Devices, Sunnyvale, CA, USA). Triplicate wells were used for each treatment.

### Cell viability

Cell viability was determined by trypan blue exclusion assay. Cells were trypsinized and resuspended in medium to achieve a homogeneous mixture. An aliquot of cell suspension was mixed with an equal volume of 0.4% trypan blue solution (Sigma). Cells were counted using a Vi-Cell cell viability analyzer (Beckman Coulter, Brea, CA, USA).

### Apoptosis assay

Cells were stained for Annexin-V and PI using the Annexin-V-Biotin Kit (Beckman Coulter) as described previously (Aird *et al*, 2010). At least 25 000 events were collected on a FACSCalibur flow cytometer (BD Biosciences, San Jose, CA, USA) and analysed using Cellquest software.

### Wound-healing assay

Cells were grown to confluence in six-well plates and then scratched with a p200 pipette tip to create a gap. The wells were rinsed with PBS to remove displaced cells and fresh media was added. For siRNA experiments, transfection of cells either with 100 nM of GLI1-specific siRNA or with 100 nM of the control siRNAs was performed as described above and 48 h after transfection, the wells were scratched. Cells were visualised using an Olympus IX51 inverted microscope. Brightfield images taken at × 40 and × 100 magnification over 24 h were analysed using the open-source CellProfiler software (Carpenter *et al*, 2006) to calculate the area of wound occupied by cells at 0 and 24 h. The percentage of wound occupied was calculated by dividing the non-recovered area at 24 h by the initial wound area at 0 h and subtracting this value as a percentage from 100%.

### Transduction with histone 2B-green fluorescent protein

SUM149 were fluorescently labelled using the BacMam-based Cellular Lights histone 2B-green fluorescent protein (H2B-GFP) reagent (Invitrogen; Cat. No 10128). SUM149 cells were seeded at 200 000 cells in a 75 cm^2^ T-flask and grown overnight as described above. Media was aspirated and the Cellular Lights transduction reagent was added. Cells were incubated in the dark for 4 h at room temperature. The transduction reagent was replaced with an enhancer reagent for 2 h and then the transduced SUM149 cells were incubated in complete media for 18 h at 37°C, 5% CO_2_.

### High-content migration assay

The H2B-GFP-labelled SUM149 cells were seeded at 10 000 cells per well in black 96-well clear bottom plates (Costar, Acton, MA, USA) and allowed to attach as described above. The cell plate was mechanically scratched using a 96-well head with p200 tips on a Biomek NX workstation (Beckman Coulter). For siRNA experiments, SUM149 cells were seeded at 3000 cells per well and incubated for 24 h before transfection either with 100 nM of GLI1-specific siRNA or with 100 nM of the negative control siRNA. Transfection was carried out as described above and 48 h after transfection, the wells were scratched with p200 tips as above. Cell plates were then imaged on a BD pathway 855 high-content imaging system (BD Biosciences) for live-cell tracking studies with environmental control (37°C, 5% CO_2_). A standard GFP filter set was used (Ex 485/20, Em 530/15) for fluorescent imaging using a Nipkow spinning-disc confocal unit to minimise photoexposure/toxicity. Tiff images (16-bit) were collected every 20 min for 24 h for each well and the data were compiled in an uncompressed avi format to yield one video file per well. The open-source CellProfiler software (Carpenter *et al*, 2006) was used for automated motility analysis. In brief, cells were identified automatically by segmenting the GFP/nuclear image and each individual cell was tracked using the TrackObjects module in CellProfiler. X, Y trajectories were tabulated for each cell observed in the time series and unique object numbers were assigned to cells. The following outputs were calculated in CellProfiler (Carpenter *et al*, 2006): cell linearity, a measure of how linear the object trajectory is during the object lifetime (calculated as (distance from initial to final location)/(integrated object distance) with value in range of [0,1]); average distance travelled, distance travelled by the object from the previous frame to the current frame (calculated as the magnitude of the distance travelled vector) and integrated distance travelled (total distance travelled by the object during the lifetime of the object) using the X,Y trajectories.

### Statistical analysis

Statistical analyses were performed using Graphpad InStat Student's two-tailed *t*-test. Differences were considered significant at *P*<0.05.

## Results

### The GLI1 transcription factor is highly expressed in the IBC cell lines SUM149 and rSUM149

As there is a growing body of evidence that shows that the downstream transcription factor GLI1 is an essential marker for Hh-pathway activation (Pasca di Magliano and Hebrok, 2003; Kasper *et al*, 2006), that sonic hedgehog (SHH) has been identified as part of a gene signature differentially regulated in IBC patients with an aggressive phenotype (Bieche *et al*, 2004) and that hyperactivation of the Hh pathway is part of an IBC gene signature (Van Laere *et al*, 2010), comparative gene expression of GLI1 was characterised in a panel of IBC and non-IBC breast cancer (MCF-7, SK-BR-3, BT474 and MDA-MB-231) cell lines. The IBC panel included the two well-established lines: SUM149 (activated ErbB1, ER/PR negative, ErbB2 low) and SUM190 (ErbB2 overexpressing, ER/PR negative), both of which were originally derived from primary IBC tumours, SUM44 (ErbB2 low, ER/PR positive) cell line derived from the pleural effusion of a patient with luminal breast cancer (Forozan *et al*, 1999), and two isogenic clonal derivatives of the SUM149 and SUM190 that have gained resistance to ErbB1/2 targeting agents such as lapatinib termed rSUM149 and rSUM190 (Aird *et al*, 2010). Phenotypic status of these cell lines is from published studies (Neve *et al*, 2006; Willmarth and Ethier, 2006; Kao *et al*, 2009; Aird *et al*, 2010; Wang *et al*, 2010b).

**Figure 1:**
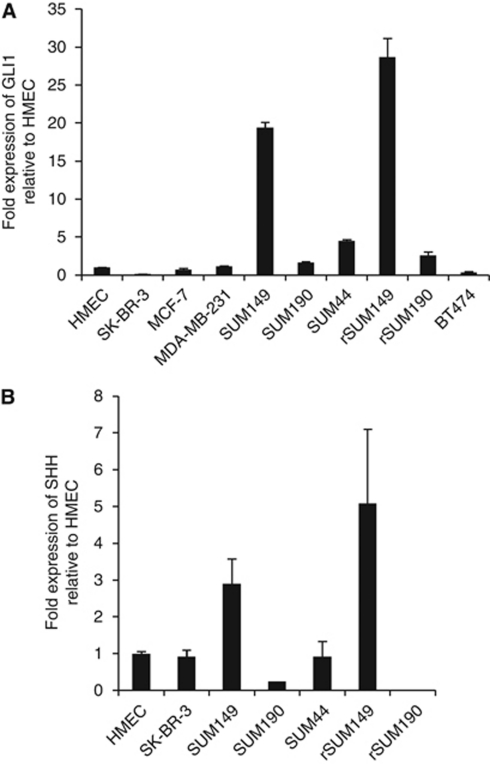
GLI1 and SHH expression in IBC and non-IBC cell lines. Total RNA (1 *μ*g) was reverse transcribed and assessed for expression of *GLI1* (**A**) and *SHH* (**B**) mRNA using real-time PCR. *β*-Actin was used as an internal control. Data indicate values performed in triplicate and the fold difference relative to HMEC.

Notably, qRT–PCR analysis of basal *GLI1* expression revealed that IBC cell lines, SUM149 and rSUM149, had a 19.4-fold and 28.7-fold higher level of *GLI1* expression, respectively, relative to HMEC, and *GLI1* expression was significantly higher compared with the other non-IBC cells tested (Figure 1A). The low expression levels of *GLI1* mRNA observed in the HMEC and MCF-7 cells are consistent with previous reports for these cell lines (Kubo *et al*, 2004; Mukherjee *et al*, 2006; Zhang *et al*, 2008). GLI1 protein levels, as assessed by immunoblot analysis, reflected *GLI1* mRNA levels in that GLI1 protein was expressed at higher levels in SUM149 (two-fold) and rSUM149 (1.5-fold) compared with MCF-7 cells and higher than in the other IBC cell lines tested, SUM190 and rSUM190 (data not shown).

### GLI1 expression in SUM149 and rSUM149 cells is Hh-ligand and SMO independent

Since *GLI1* mRNA levels are reliable indicators of Hh-pathway activation (Kasper *et al*, 2006), our data suggest that the Hh pathway may be constitutively active in SUM149 and rSUM149 cells, resulting in the observed high expression levels of GLI1. Hh-pathway activation generally proceeds through one of two main mechanisms: either Hh-ligand-dependent or Hh-ligand-independent (Rubin and de Sauvage, 2006). We first investigated whether GLI1 activation in SUM149 and rSUM149 cells occurs through a Hh-ligand-dependent mechanism by analysing whether these cells express sonic hedgehog (SHH) ligand. qRT–PCR analysis of basal *SHH* ligand expression revealed an ∼3-fold and ∼5-fold higher level of *SHH* expression in SUM149 and rSUM149 cells, respectively, compared with HMEC and the other IBC and non-IBC cells tested (Figure 1B).

**Figure 2:**
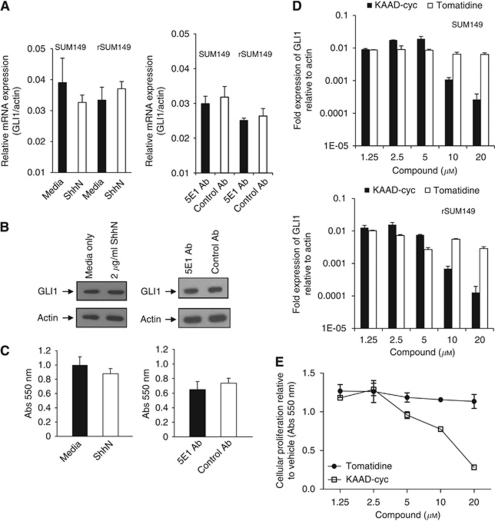
GLI1 expression in SUM149 cells is Hh-ligand and SMO independent. (**A**) RT–PCR analysis of *GLI1* mRNA expression in SUM149 and rSUM149 cells treated for 72 h with media or 2 *μ*g ml^–1^ recombinant ShhN (left panel) or with 100 *μ*g ml^–1^ 5E1 antibody or 100 *μ*g ml^–1^ isotype control antibody (right panel). *β*-Actin was used as an internal control. (**B**) Immunoblot analysis for GLI1 protein expression in SUM149 cells (7 × 10^4^ cells per well in six-well plates) treated for 72 h with media or ShhN (left panel) or with 5E1 antibody or control antibody (right panel). Membranes were stripped and reprobed for *β*-actin as an internal control for equal loading. (**C**) Cell proliferation was determined by MTT assay in SUM149 cells (2000 cells per well in 96-well plates) treated for 72 h with media or ShhN (left panel) or with 5E1 antibody or control antibody (right panel). (**D**) RT–PCR analysis of *GLI1* mRNA expression in SUM149 (upper panel) and rSUM149 (lower panel) cells treated for 72 h with the indicated concentrations of KAAD-cyclopamine (KAAD-cyc) or its inactive analogue tomatidine. *β*-Actin was used as an internal control. (**E**) Cell proliferation was determined by MTT assay in SUM149 cells (2000 cells per well) plated in 96-well plates following treatment for 72 h with the indicated concentrations of KAAD-cyc and its inactive analogue tomatidine.

We next tested the responsiveness of these IBC cell lines to exogenous ShhN (active N-terminal form of Shh) ligand. Using RT–PCR of GLI1 mRNA levels as a sensitive readout of Hh-pathway activity, we observed that the addition of exogenous recombinant ShhN ligand to SUM149 or rSUM149 cells compared with media alone did not increase the expression of GLI1 (Figure 2A, left panel).

To further investigate any role for autocrine Hh-ligand-dependent growth, SUM149 and rSUM149 cells were cultured with a pan-Hh neutralising monoclonal antibody (5E1) (Ericson *et al*, 1996). 5E1 did not significantly reduce the expression of GLI1 mRNA in SUM149 or rSUM149 cells treated with the anti-Hh mAb compared with an isotype control (Figure 2A, right panel). In addition, neither the addition of ShhN protein nor 5E1 Ab had any effect on GLI1 protein levels compared with controls as assessed by immunoblot (Figure 2B) and did not affect SUM149 cell proliferation (Figure 2C). These results suggest that although SHH is expressed in SUM149 and rSUM149 cells, GLI1 activation does not appear to be Hh-ligand-dependent.

Hh-ligand-independent activation of GLI1 expression via loss of *PTCH* or oncogenic mutations in *SMO* have been reported for a number of cancers and pharmacological inhibition with SMO-directed inhibitors shown to block Hh signalling and cell proliferation (Taipale *et al*, 2000; Chen *et al*, 2002). We tested the effects of the SMO inhibitor KAAD-cyclopamine (KAAD-cyc) compared with an inactive analogue (tomatidine) on GLI1 mRNA levels and observed that concentrations of 10 *μ*M or greater of KAAD-cyc were required to reduce GLI1 levels in SUM149 or rSUM149 cells (Figure 2D). A comparable response was observed when assessing the effects of KAAD-cyc on SUM149 (Figure 2E) and rSUM149 (data not shown) cell proliferation as measured by MTT assay. Using the same stock solution of KAAD-cyc in the Hh responsive C3H10T1/2 cell assay (Williams *et al*, 1999), we determined IC_50_ values of ∼10 nM for KAAD-cyclopamine (data not shown) and comparable values have been reported in other Hh reporter cell assays (Taipale *et al*, 2000). Hence, the micromolar concentration of KAAD-cyc required to block SUM149 cell proliferation here is much higher than that typically required to inhibit Hh-pathway activity, suggesting a SMO-independent off-target inhibitory effect as has been suggested for its *in vitro* growth inhibitory effects on other cancer cell lines (Yauch *et al*, 2008; Zhang *et al*, 2008). As of yet, no other non-SMO targets have been identified for KAAD-cyclopamine (Zhang *et al*, 2008).

### Downregulation of GLI1 expression in SUM149 by either siRNA or a GLI-directed inhibitor results in decreased cell growth

**Figure 3:**
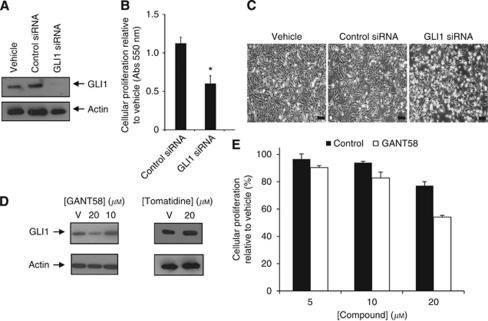
Reducing GLI1 expression in SUM149 cells by either siRNA knockdown or small molecule treatment results in decreased proliferation. SUM149 cells (7 × 10^4^ cells per well in six-well plates) were transfected with 100 nM of GLI1 siRNA or control siRNA for 72 h. Vehicle is lipofectamine alone. (**A**) Immunoblot analysis of GLI1 protein expression. The blot was stripped and reprobed for *β*-actin as an internal control for equal loading. (**B**) Cell proliferation was measured after 72 h by MTT assay. Experiments were performed in triplicate, *P*-value <0.05. (**C**) SUM149 cells were examined for morphology under phase light microscopy using an Olympus IX51 inverted microscope at a × 100 magnification. Bar=100 *μ*m. (**D**) Immunoblot for GLI1 protein expression in SUM149 cells treated for 72 h with indicated concentrations of GANT58 (left panel) or tomatidine (right panel). *β*-Actin was used as an internal control for equal loading. (**E**) Cell proliferation was measured by MTT for SUM149 cells following treatment for 72 h with indicated concentrations of GANT58 or tomatidine as a control. Data are shown as relative to control (%).

Since SUM149 and rSUM149 cells express high levels of GLI1 but these high levels appear to be Hh-ligand and SMO independent, we evaluated the effects of direct silencing of GLI1 expression on cell behaviour by transfecting GLI1 siRNA and control siRNA for 72 h. Immunoblot analysis in Figure 3A showed decreased GLI1 protein expression by 93% at 100 nM GLI1 siRNA compared with control siRNA (*P*-value <0.05). Further, a significant decrease (*P*<0.05) in cell proliferation was observed in GLI1 siRNA-treated cells (46–50% decrease compared with controls, Figure 3B) as assessed by the MTT assay. Phase-contrast analysis showed a higher degree of nucleation in the SUM149 cells transfected with GLI1 siRNA compared with the vehicle (lipofectamine only) and control siRNA-transfected cells (Figure 3C).

In addition, we compared a recently described direct inhibitor of GLI1 transcription (GANT58) that acts downstream of Hh signalling (Lauth *et al*, 2007). Immunoblot analysis of SUM149 cells after treatment with GANT58 for 72 h showed GLI1 protein levels decreased at 20 *μ*M compared with untreated cells (Figure 3D, left panel) or cells treated with 20 *μ*M tomatidine (Figure 3D, right panel). Characterisation of cell proliferation using an MTT assay revealed that proliferation of SUM149 cells was significantly suppressed at 20 *μ*M GANT58 (54% proliferation *vs* 80% for control) (Figure 3E). GANT58 was effective on SUM149 cells at concentrations comparable to those we determined in the C3H10T1/2 assay (data not shown) and to published IC_50_ values of 5 *μ*M for GANT58 in GLI reporter assays (Lauth *et al*, 2007), suggesting that it is acting on GLI.

As silencing with siRNA was a more effective method at reducing GLI1 levels in SUM149 cells compared with the small molecule GANT58, subsequent studies were done with siRNA.

### GLI1 silencing induces apoptosis

**Figure 4:**
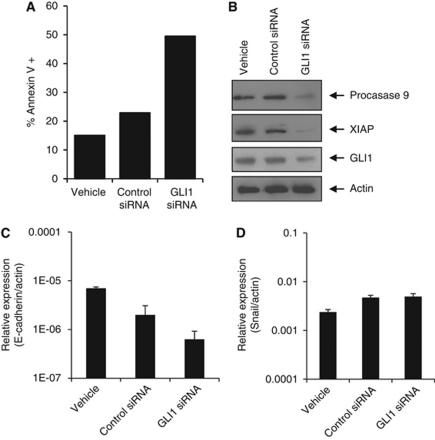
GLI1 siRNA knockdown in SUM149 cells results in increased apoptosis and decreased levels of anti-apoptotic proteins. (**A**) Apoptosis was determined by the Annexin-V/PI assay for SUM149 cells (7 × 10^4^ cells per well) plated in six-well plates and transfected with 100 nM of control or GLI1 siRNA for 72 h. (**B**) Immunoblots of procaspase 9, XIAP and GLI1 protein expression in SUM149 cells (7 × 10^4^ cells in six-well plates) transfected with 100 nM of GLI1 siRNA or control siRNA for 72 h. The blots were stripped and reprobed for *β*-actin as an internal control for equal loading. *E-cadherin* (**C**) and *Snail* (**D**) mRNA expression levels were assessed by RT–PCR in SUM149 cells transfected with 100 nM of GLI1 siRNA or 100 nM control siRNA for 72 h. Vehicle is liptofectamine alone treated cells. *β*-Actin was used as an internal control and data indicate values performed in triplicate and the fold difference relative to *β*-actin.

To determine if the observed decrease in proliferation seen with GLI1 silencing was due to an increase in apoptosis, Annexin-V staining was performed. Both early apoptotic cells (Annexin-V positive and PI negative) and late apoptotic cells (Annexin-V positive and PI positive) were counted. Data in Figure 4A demonstrate that after 72 h, GLI1 siRNA treated SUM149 cells had a 3.2-fold increase in Annexin-V-positive cells (58%) over the control siRNA-transfected cells (18%). To further understand the mechanism of the observed cell death, immunoblot analysis was conducted to examine the effect of GLI1 knockdown on the expression of key apoptotic signalling proteins. Immunoblot analysis (Figure 4B) shows that at 72 h, GLI1 siRNA dramatically decreased the expression of procaspase 9 compared with control siRNA. This corresponded with a significant decrease in expression of XIAP (Figure 4B), one of the most potent caspase inhibitors and anti-apoptotic proteins that has recently been reported to be a key determinant in apoptotic dysregulation in IBC (Aird *et al*, 2008, 2010).

In light of the reported high levels of E-cadherin associated with the IBC invasive phenotype and in SUM149 cells (Kleer *et al*, 2001; Colpaert *et al*, 2003; Hoffmeyer *et al*, 2005; Dong *et al*, 2007), we were interested to see what affect GLI1 silencing would have on E-cadherin expression in SUM149. A mechanistic link between GLI1 and E-cadherin occurs as part of epithelial transformation via the transcription factor Snail (Li *et al*, 2006, 2007). We found that GLI1 silencing in SUM149 cells reduced E-cadherin levels as assessed by qRT–PCR compared with controls (Figure 4C) but did not affect Snail levels (Figure 4D).

### GLI1 silencing impairs IBC cell migration

Since targeting GLI1 inhibited cell proliferation, increased apoptosis and decreased E-cadherin that has been identified to be maintained in aggressive IBC phenotype, we investigated the effect of GLI1 silencing on the migration potential of SUM149 and rSUM149 cells using an *in vitro* wound-healing assay. The *in vitro* wound-healing assay is frequently used as a simple assay to mimic and assess migration (Liang *et al*, 2007) and has been used previously to assess the invasiveness of SUM149 cells (Hoffmeyer *et al*, 2005). In this assay, an artificial wound is created by scratching a confluent monolayer of cells and the ability of the cells to move into and close the wound is thought to predict their migration ability *in vivo* (Liang *et al*, 2007).

**Figure 5:**
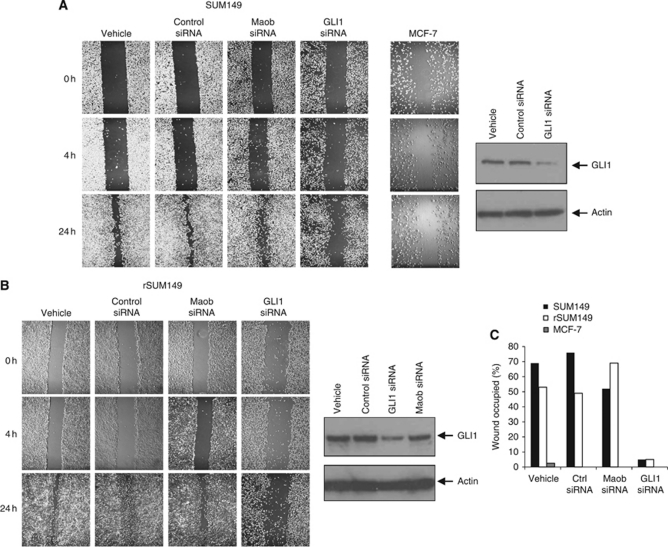
Silencing GLI1 expression impairs motility of SUM149 and rSUM149 cells. Cells were grown to confluence in six-well plates and scratched with a sterile pipette tip. Closure of the wound was monitored over 24 h. *In vitro* wound-healing assay on SUM149 (**A**) and rSUM149 cells (**B**) treated with vehicle (lipofectamine alone), control siRNA, Maob siRNA and GLI1 siRNA. Images were taken at × 40 magnification over 24 h. Wound-healing assay for MCF-7 is shown as a control. Immunoblots are shown to confirm significant knockdown of GLI1 protein only in cells treated with siRNA to *GLI1*. (**C**) Images from (**A** and **B**) were analysed using CellProfiler software to calculate the area occupied by cells at 0 and 24 h. The percentage of wound occupied was calculated by dividing the non-recovered area at 24 h by the initial wound area at 0 h and subtracting this value as a percent from 100%.

In our study, cells were seeded into six-well plates, grown to confluence and scratched with a p200 pipette tip to create a wound. Migration of cells into the wound was assessed by taking phase-contrast images at time points over 24 h. MCF-7 cells, a breast cancer cell line with low invasive potential (Neve *et al*, 2006), showed little migration in the wound-healing assay over 24 h (Figure 5A). In contrast, SUM149 (Figure 5A, vehicle) and rSUM149 cells (Figure 5B, vehicle) migrate into and close the ‘wound’ over the same 24 h time period consistent with previous observations that SUM149 cells are more invasive than MCF-7 cells (Neve *et al*, 2006).

We next tested the effect of silencing GLI1 expression in SUM149 and rSUM149 cells on their migratory phenotype. Cells were first transfected with either GLI1 siRNA, siRNA to an unrelated target Maob or control siRNA for 48 h and then scratched as before. Compared with both vehicle-treated (lipofectamine alone), control and Maob siRNA-treated cells, silencing of GLI1 in SUM149 (Figure 5A) and rSUM149 (Figure 5B) cells resulted in a significant reduction in migration ability. Cell viability was also assessed after siRNA treatment and subsequent wounding for 24 h, and viability was only marginally decreased by ∼20% in GLI1 siRNA-treated SUM149 cells compared with vehicle and control siRNA treatment. Comparable values were observed for rSUM149 cells (data not shown).

To quantify SUM149 cell motility in our scratch assays, the brightfield images taken at × 40 magnification shown in Figures 5A and B were analysed using the open-source CellProfiler software (Carpenter *et al*, 2006) to calculate the area occupied by cells at 0 and 24 h. The percentage of wound area recovered was calculated by dividing the non-recovered area at 24 h by the initial wound area at 0 h. This analysis (Figure 5C) showed that 24 h after wounding, the vehicle and control siRNA-treated SUM149 cells had occupied 68 and 75%, respectively, of the wound while the GLI1 siRNA SUM149 cells had occupied only ∼5%, a value comparable to that determined for MCF-7. Comparable data were obtained for rSUM149 cells (Figure 5C). These results suggest that silencing GLI1 in SUM149 and rSUM149 cells has a significant effect on cell motility. We also tested the effect of GANT58 (5, 10 and 20 *μ*M) on SUM149 motility and observed a modest reduction in migration at the highest dose compared with controls (data not shown).

### High-content imaging analysis of H2B-GFP-tagged SUM149 cells shows that silencing GLI1 reduces cell distance travelled and linearity of cell motility

High-content automated microscopy (HCAM) was used to generate quantitative information from the wound-healing assay, and to extract other critical parameters defining migration behaviour. High-content automated microscopy was conducted using the BD pathway 855 imaging system, which is multi-well plate compatible and equipped with an environmentally controlled chamber (CO_2_ and temperature), allowing live-cell imaging over many hours. For wounding, cells were seeded in 96-well plates and after reaching confluence scratched simultaneously using p200 tips on a 96-well head configured on a Biomek NX robotics workstation.

**Figure 6:**
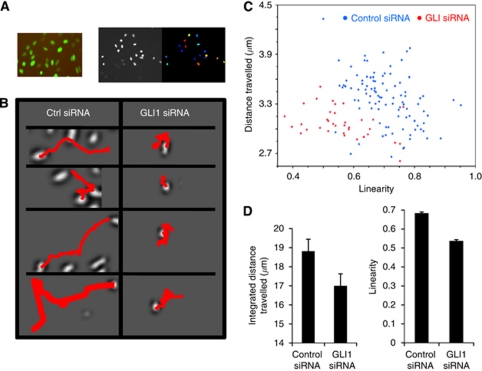
High-content imaging and migration analysis of SUM149 H2B-GFP cells demonstrate that cell linearity and distance travelled are decreased in SUM149 cells with GLI1 attenuated. (**A**) (Left panel) SUM149 cells transduced with BacMam H2B-GFP viral particles. Image was taken with a × 10 Olympus objective. (Right panel) Automated cell detection was performed using CellProfiler by segmenting each nucleus using the Otsu image segmentation. Segmented nuclei are shown by colour (right image). (**B**) Cell plates were imaged on a BD pathway 855 high-content imaging system with Nikpow spinning disc for live-cell fluorescent imaging. Tiff images (16-bit) were collected every 20 min for 24 h for each well and the data were compiled in an uncompressed avi format to yield one video file per well. CellProfiler software was used for video tracking using the minimum displacement method in the TrackObjects module. Representative trajectories for SUM149 cells treated with a negative control siRNA or GLI1 siRNA are shown. (**C**) X, Y trajectories were tabulated for each cell observed in the time series and unique object numbers were assigned to cells. Population scatterplot showing distance travelled *vs* linearity for cells treated with GLI1 siRNA or negative control siRNA. (**D**) (Left panel) Integrated distance travelled for the negative control siRNA and GLI1 siRNA-treated samples. (Right panel) Average linearity for the negative control siRNA and GLI1 siRNA-treated samples.

SUM149 cells were transduced with baculoviral particles expressing H2B-GFP to label the nuclei and Figure 6A (left panel) reveals the live-cell tracking as imaged on the BD Imaging workstation. The advantages of H2B-GFP labelling are that this reporter system does not affect cell viability (Quaranta *et al*, 2009) and cells are brightly labelled by tagging nuclei compared with diffuse labelling as for cytoplasmic markers. As part of our preliminary high-content studies, we observed that dyes such as MitoTracker Red and CellTracker were not suitable for tracking purposes as SUM149 cells treated with these dyes exhibited no motility (data not shown). Further, extended live-cell fluorescent imaging can cause significant phototoxicity so we chose to limit overall light exposure by using a spinning-disc confocal unit, rather than widefield. Although exposure times increase, the spinning disc reduces the field of illumination from the entire well to pinhole sized, dramatically reducing the overall photoexposure per cell.

High-content imaging of H2B-GFP SUM149 cells after GLI1 silencing allowed us to measure effects on multiple motility parameters through careful feature extraction from X,Y trajectories. SUM149 cells were seeded in 96-well plates and after reaching confluence, silenced with GLI1 siRNA and mechanically scratched as described above. Migration of the cells into the wound was assessed over 24 h and image data were filtered, cells segmented and tracked using both NIH ImageJ and CellProfiler (Figure 6A, right panel) for each well per condition. The live-cell tracking algorithm for the vehicle control (lipofectamine only) and negative control siRNA showed similar motility, whereas the motility of GLI1-silenced SUM149 cells was low. Many features of each cellular trajectory were tabulated and compared for each experimental condition. Representative trajectories for SUM149 treated with either the negative control siRNA or GLI1 siRNA (Figure 6B) shows that the SUM149 cells with silenced GLI1 have reduced translational progress. X, Y trajectories were tabulated for each cell observed in the time series and unique object numbers were assigned to cells. We extracted numerous features from the X, Y trajectories and found significant changes in the integrated distance travelled, and the calculated linearity of the trajectory. A population scatterplot (Figure 6C) shows distance travelled *vs* linearity for individual cells from both the GLI1 knockdown (red) and the negative control siRNA-treated SUM149 cells (blue). Although there is some overlap between the two cell sets with a small number of GLI1 siRNA cells still showing high motility, there is a dramatic difference between the control and siRNA-treated SUM149 cells. Plotting these data show a significant decrease in both distance travelled (Figure 6D, left panel) and linearity (Figure 6D, right panel) in the GLI1 knockdown compared with control siRNA-treated SUM149 cells, suggesting a link between GLI1 expression and cell motility.

## Discussion

We report herein a role for the transcription factor GLI1 in the growth, survival and migration of two IBC cell lines, SUM149 cells, a triple-negative IBC cell line with activated EGFR, isolated from a primary tumour of an IBC patient (Forozan *et al*, 1999; Ignatoski and Ethier, 1999) and rSUM149, an isogenic cell line with acquired resistance to ErbB1/2 inhibitors (Aird *et al*, 2010). Both SUM149 and rSUM149 expressed significantly higher levels of GLI1 compared with other IBC cell lines SUM190 and rSUM190, non-IBC cell lines MCF-7, SK-BR-3, BT474 MDA-MB-231 and normal mammary epithelial cells evaluated in this study. To validate the role of elevated GLI1 expression in SUM149 and rSUM149 cell growth, we investigated direct blocking of GLI1 expression by RNA interference. The decrease in cell proliferation and increase in apoptosis in SUM149 cells treated with GLI1 siRNA corresponded with decreased levels of the most potent anti-apoptotic proteins XIAP and its target procaspase 9. The role of apoptotic dysregulation as mediated by the inhibitor of apoptosis protein XIAP has been recently reported by our group as a mechanism of therapeutic resistance to apoptosis in IBC cells (Aird *et al*, 2008, 2010). Further, herein we observed that the migration ability of the SUM149 and rSUM149 cells when GLI1 was silenced was dramatically reduced compared with control-transfected cells as assessed in both a conventional scratch assay and a novel automated wound-healing assay utilising H2B-GFP-tagged cells. The present study, therefore suggests that upregulation of GLI1 is important for survival and migration in these IBC cells.

The only previous reports connecting the Hh-Gli pathway with IBC was a gene array study linking a three-gene signature including the *Sonic hedgehog* (*SHH*) gene with poor outcome in IBC patients (Bieche *et al*, 2004), and another gene expression study demonstrating that IBC is characterised by Hh-pathway hyperactivation (Van Laere *et al*, 2010). GLI1 has been reported extensively as a universal marker for Hh-pathway activation. Elevated GLI1 expression and its role in a number of cancers has been widely reported, including a recent study that the conditional expression of GLI1 in mouse mammary glands results in mammary tumours (Fiaschi *et al*, 2009). Aberrant activation of GLI1 expression might arise from a number of possible mechanisms with both canonical Hh-pathway-dependent (Rubin and de Sauvage, 2006; Yauch *et al*, 2008) and non-canonical Hh-independent (Lauth and Toftgard, 2007; Jenkins, 2009) activation of GLI1 reported for different cancers. We observed that neither the addition of exogenous Shh ligand nor a blocking Hh antibody was able to affect the growth or levels of GLI1 in SUM149 or rSUM149 IBC cell lines. Lack of responsiveness to added Hh-ligand is expected considering the very low levels of SMO expression observed in SUM149 cells (data not shown). Similarly, most non-IBC breast cancer cell lines do not respond to exogenous ShhN to activate Hh signalling or increase GLI (Zhang *et al*, 2008). Taken together with our observation of the high concentration of KAAD-cyc required to elicit growth effects, the present study suggests that in SUM149 cells GLI1 activation is not Hh-ligand or SMO dependent.

The data from GLI1 siRNA silencing studies suggest a more effective approach to targeting SUM149 cells would be by directly inhibiting GLI1 transcription. To further probe the molecular basis for GLI1 activation in SUM149 cells, we tested the effects of a direct GLI inhibitor, GANT58, which has been reported to block GLI1-induced transcription *in vitro* and prevent additional tumour growth in xenograft tumour models (Lauth *et al*, 2007). We observed that GANT58 also reduced cell proliferation of SUM149 cells and reduced GLI1 protein expression. Although its mechanism of action is unclear, GANT58 has been reported to act on nuclear localised GLI (Lauth *et al*, 2007) and it appears to be highly selective for GLI as it has no effect on other signalling pathways such as TNF/NFκB and Ras/MAPK or on protein folding or nuclear transport (Lauth *et al*, 2007). Targeting GLI1 in the clinic with siRNA is not currently feasible and the availability of more potent GLI inhibitors would be beneficial. Other GLI1 inhibitors have been recently described (Hosoya *et al*, 2008; Hyman *et al*, 2009; Mahindroo *et al*, 2009) that target multiple steps in GLI regulation, although with micromolar potencies comparable to the GANT compound.

IBC is an aggressive clinical subtype of breast cancer that is an extremely invasive and metastatic disease (Cristofanilli *et al*, 2007) that falls into two major subtypes; Basal-like or ErbB2 overexpressing (Van Laere *et al*, 2007). The cellular models that were used for this study are the only well-established IBC cellular models available; SUM149, an ErbB1 activated, triple-negative (ErbB2-, ER- and PR-) cell line and SUM190, an ErbB2 overexpressed, ER- cell line, both of which have been derived from primary IBC tumours (Forozan *et al*, 1999). Both these cell lines are sensitive to ErbB1/2 targeting agents (Aird *et al*, 2008, 2010). SUM149 cells were generated from an aggressive IBC (Forozan *et al*, 1999) and have been characterised as a Basal B subtype of breast cancer cell (Neve *et al*, 2006), which is frequently more aggressive and invasive than other breast cancer cell types. As GLI1 has been observed to promote cell migration and invasion in other cancer cell types (Das *et al*, 2009; Lo *et al*, 2009), including non-IBC breast cancer cell lines (Kameda *et al*, 2009; Souzaki *et al*, 2011), we were interested to determine if it has a role in the invasive potential of SUM149 and rSUM149 cells. Further, IBC is unusual in that it is an aggressive type of breast cancer that maintains strong E-cadherin expression (Kleer *et al*, 2001; Colpaert *et al*, 2003; Dong *et al*, 2007). We wanted to investigate whether the high levels of GLI1 in SUM149 cells have a role in its unique migratory phenotype. Silencing of GLI1 reduced the level of E-cadherin but not of Snail, a transcription factor that promotes epithelial–mesenchymal transitions and is a key target of GLI1 (Li *et al*, 2006, 2007). The molecular mechanisms linking GLI1 expression to E-cadherin and the invasive phenotype of SUM149 warrants further investigation.

In our study, we used the *in vitro* wound-healing assay as a measure of migration and found that silencing of GLI1 in SUM149 and rSUM149 cells dramatically reduces cell migration over 24 h. From a previous study looking at the invasive potential of 30 breast cancer cell lines across subtypes, SUM149 falls within the Basal B subtype of highly invasive cells as assessed in 24 h Boyden chamber assays (Neve *et al*, 2006). In contrast, other reports have suggested a less migratory phenotype for SUM149 (Nieman *et al*, 1999; Hoffmeyer *et al*, 2005) and that migration occurs by a passive dissemination process (Hoffmeyer *et al*, 2005). These differences may be attributable to differences in format and time assessed as one report used the wound-healing assay over a shorter (7.5 h) time frame (Hoffmeyer *et al*, 2005).

Studies in non-IBC breast cancer cell lines have suggested a link between ER status, GLI1 levels and survival, and shown that GLI1 silencing in ER negative breast cancer markedly reduces survival (Zhao *et al*, 2009; Xu *et al*, 2010) and invasiveness (Kameda *et al*, 2009; Souzaki *et al*, 2011). Although both SUM149 and SUM190 are ER- we only observed high GLI1 levels in SUM149. Presumably additional factors contribute to the elevated GLI1 status in the triple-negative Basal B-like SUM149 cells.

In addition, we were interested in developing high-content imaging assays to not only glean more quantitative information from the motility wound-healing assay, but to also extract other critical parameters defining migration behaviour. The advantages of the wound-healing assay are that it can be miniaturised and is compatible with microscopy, making it adaptable to high-throughput imaging. Such a HCAM system allows the analysis of both population and single-cell data on a heterogeneous population of cells and can potentially link phenotypic variability to genetic differences (Quaranta *et al*, 2009). To obtain a more quantitative assessment and further assess the role of GLI1 in the migratory phenotype, we tagged SUM149 cells with H2B-GFP to fluorescently label nuclei of living cells and using a high-content live-cell fluorescence-based imaging system demonstrated that we can track not only cell migration but additional parameters of motility such as linearity of movement and integrated distance travelled. Both average linearity and distance travelled were reduced in SUM149 cells with GLI1 silenced. Further, our proof-of-principle study described here allowed us to distinguish differences in phenotype. GLI1 knockdown cells continue to move although with much reduced linearity and distance travelled. Indeed, the mean velocity between controls and GLI1 knockdown did not change significantly. In comparison, we observed that dyes such as MitoTracker Red affect the motility phenotype of SUM149 cells with the result that these cells have no motility. We expect this type of multi-parameter analysis of cell migration after H2B-GFP tagging to be generally applicable to many cell types to assess effects on migration.

Our data suggest a Hh-ligand-independent/SMO-independent mechanism of GLI1 gene overexpression operates in SUM149 and rSUM149 cells although we cannot rule out a role for other Hh-pathway components such as changes in SUFU (suppressor of FUSED) in activating GLI1. A number of other pathways have been reported to influence the cellular levels of GLI1 (Riobo *et al*, 2006b) including TGF-*β* (Dennler *et al*, 2007), PI3K/AKT (Riobo *et al*, 2006a), Ras/Mek (Stecca *et al*, 2007), and a recently described novel GLI1-CDK2-dependent mechanism (Rizvi *et al*, 2010). Further analyses using a range of pharmacological inhibitors to assess the role of other pathways in activating GLI1 and the phenotype of SUM149 and rSUM149 cells are planned in future studies. The understanding of those pathways converging on GLI in SUM149 will guide drug development strategies. It has been suggested that due to the complexity of signalling inputs into GLI (Ruiz *et al*, 2007; Stecca and Ruiz, 2010), targeting GLI in cancers may provide a more comprehensive strategy for treating both canonical and non-canonical Hh-pathway-dependent cancers. Further, it has been proposed that GLI-directed compounds that block growth in one cell type may be ineffective in others (Hyman *et al*, 2009) and indeed, it may be necessary to directly screen specific cancer cell lines for compounds that specifically inhibit GLI1 expression in a particular cellular context.

Few studies have examined the molecular biology of IBC due to its relative low incidence; however, due to its high aggressiveness, mortality rate, and resistance to chemotherapeutic drugs (Rouesse *et al*, 1986), additional investigations are warranted. In light of our observation that GLI1 has a role in proliferation, survival and migration of a subset of IBC cells, we propose that direct targeting of GLI1 transcription may be a novel and promising strategy for targeting triple-negative/Basal B IBC modelled by SUM149 cells.

## References

[bib1] Aird KM, Ding X, Baras A, Wei J, Morse MA, Clay T, Lyerly HK, Devi GR (2008) Trastuzumab signaling in ErbB2-overexpressing inflammatory breast cancer correlates with X-linked inhibitor of apoptosis protein expression. Mol Cancer Ther 7: 38–47 18202008 10.1158/1535-7163.MCT-07-0370

[bib2] Aird KM, Ghanayem RB, Peplinski S, Lyerly HK, Devi GR (2010) X-linked inhibitor of apoptosis protein inhibits apoptosis in inflammatory breast cancer cells with acquired resistance to an ErbB1/2 tyrosine kinase inhibitor. Mol Cancer Ther 9: 1431–1442 10.1158/1535-7163.MCT-10-0160 PMC2957814 20406946

[bib3] Bieche I, Lerebours F, Tozlu S, Espie M, Marty M, Lidereau R (2004) Molecular profiling of inflammatory breast cancer: identification of a poor-prognosis gene expression signature. Clin Cancer Res 10: 6789–6795 15501955 10.1158/1078-0432.CCR-04-0306

[bib4] Carpenter A, Jones T, Lamprecht M, Clarke C, Kang I, Friman O, Guertin D, Chang J, Lindquist R, Moffat J, Golland P, Sabatini D (2006) CellProfiler: image analysis software for identifying and quantifying cell phenotypes. Genome Biol 7: R100 17076895 10.1186/gb-2006-7-10-r100 PMC1794559

[bib5] Charafe-Jauffret E, Tarpin C, Bardou VJ, Bertucci F, Ginestier C, Braud AC, Puig B, Geneix J, Hassoun J, Birnbaum D, Jacquemier J, Viens P (2004) Immunophenotypic analysis of inflammatory breast cancers: identification of an ‘inflammatory signature’. J Pathol 202: 265–273 14991891 10.1002/path.1515

[bib6] Chen JK, Taipale J, Cooper MK, Beachy PA (2002) Inhibition of Hedgehog signaling by direct binding of cyclopamine to Smoothened. Genes Dev 16: 2743–2748 12414725 10.1101/gad.1025302 PMC187469

[bib7] Chen X, Horiuchi A, Kikuchi N, Osada R, Yoshida J, Shiozawa T, Konishi I (2007) Hedgehog signal pathway is activated in ovarian carcinomas, correlating with cell proliferation: its inhibition leads to growth suppression and apoptosis. Cancer Sci 98: 68–76 17083567 10.1111/j.1349-7006.2006.00353.x PMC11158570

[bib8] Chinchilla P, Xiao L, Kazanietz M, Riobo N (2010) Hedgehog proteins activate pro-angiogenic responses in endothelial cells through non-canonical signaling pathways. Cell Cycle 9: 570–579 20081366 10.4161/cc.9.3.10591

[bib9] Colpaert CG, Vermeulen PB, Benoy I, Soubry A, van Roy F, van Beest P, Goovaerts G, Dirix LY, van Dam P, Fox SB, Harris AL, van Marck EA (2003) Inflammatory breast cancer shows angiogenesis with high endothelial proliferation rate and strong E-cadherin expression. Br J Cancer 88: 718–725 12618881 10.1038/sj.bjc.6600807 PMC2376338

[bib10] Cristofanilli M, Valero V, Buzdar A, Kau S, Broglio K, Gonzalez-Angulo A, Sneige N, Islam R, Ueno N, Buchholz T (2007) Inflammatory breast cancer (IBC) and patterns of recurrence: understanding the biology of a unique disease. Cancer 110: 1436–1444 17694554 10.1002/cncr.22927

[bib11] Das S, Harris L, Metge B, Liu S, Riker A, Samant R, Shevde L (2009) The Hedgehog pathway transcription factor GLI1 promotes malignant behavior of cancer cells by up-regulating osteopontin. J Biol Chem 284: 22888–22897 19556240 10.1074/jbc.M109.021949 PMC2755696

[bib12] Dennler S, André J, Alexaki I, Li A, Magnaldo T, Ten Dijke P, Wang X, Verrecchia F, Mauviel A (2007) Induction of Sonic hedgehog mediators by transforming growth factor-{beta}: Smad3-dependent activation of Gli2 and Gli1 expression *in vitro* and *in vivo*. Cancer Res 67: 6981–6986 17638910 10.1158/0008-5472.CAN-07-0491

[bib13] Dong HM, Liu G, Hou YF, Wu J, Lu JS, Luo JM, Shen ZZ, Shao ZM (2007) Dominant-negative E-cadherin inhibits the invasiveness of inflammatory breast cancer cells *in vitro*. J Cancer Res Clin Oncol 133: 83–92 16932944 10.1007/s00432-006-0140-6 PMC12160849

[bib14] Ericson J, Morton S, Kawakami A, Roelink H, Jessell T (1996) Two critical periods of Sonic Hedgehog signaling required for the specification of motor neuron identity. Cell 87: 661–674 8929535 10.1016/s0092-8674(00)81386-0

[bib15] Feng YZ, Shiozawa T, Miyamoto T, Kashima H, Kurai M, Suzuki A, Ying-Song J, Konishi I (2007) Overexpression of hedgehog signaling molecules and its involvement in the proliferation of endometrial carcinoma cells. Clin Cancer Res 13: 1389–1398 17332280 10.1158/1078-0432.CCR-06-1407

[bib16] Fernandez-Zapico M (2008) Primers on molecular pathways GLI: more than just Hedgehog? Pancreatology 8: 227–229 18497536 10.1159/000134271 PMC2718430

[bib17] Fiaschi M, Rozell B, Bergstrom A, Toftgard R (2009) Development of mammary tumors by conditional expression of GLI1. Cancer Res 69: 4810–4817 19458072 10.1158/0008-5472.CAN-08-3938 PMC2689922

[bib18] Forozan F, Veldman R, Ammerman CA, Parsa NZ, Kallioniemi A, Kallioniemi O-P, Ethier SP (1999) Molecular cytogenetic analysis of 11 new breast cancer cell lines. Br J Cancer 81: 1328–1334 10604729 10.1038/sj.bjc.6695007 PMC2362964

[bib19] Hance K, Anderson W, Devesa S, Young H, Levine P (2005) Trends in inflammatory breast carcinoma incidence and survival: the surveillance, epidemiology, and end results program at the National Cancer Institute. J Natl Cancer Inst 97: 966–975 15998949 10.1093/jnci/dji172 PMC2844937

[bib20] Hoffmeyer MR, Wall KM, Dharmawardhane SF (2005) *In vitro* analysis of the invasive phenotype of SUM 149, an inflammatory breast cancer cell line. Cancer Cell Int 5: 11 15857504 10.1186/1475-2867-5-11 PMC1090601

[bib21] Hosoya T, Arai M, Koyano T, Kowithayakorn T, Ishibashi M (2008) Naturally occurring small-molecule inhibitors of hedgehog/GLI-mediated transcription. Chembiochem 9: 1082–1092 18357592 10.1002/cbic.200700511

[bib22] Hyman J, Firestone A, Heine V, Zhao Y, Ocasio C, Han K, Sun M, Rack P, Sinha S, Wu J, Solow-Cordero DE, Jiang J, Rowitch DH, Chen JK (2009) Small-molecule inhibitors reveal multiple strategies for Hedgehog pathway blockade. Proc Natl Acad Sci USA 106: 14132–14137 19666565 10.1073/pnas.0907134106 PMC2721821

[bib23] Ignatoski K, Ethier S (1999) Constitutive activation of pp125fak in newly isolated human breast cancer cell lines. Breast Cancer Res Treat 54: 173–182 10424408 10.1023/a:1006135331912

[bib24] Inaguma S, Kasai K, Ikeda H (2011) GLI1 facilitates the migration and invasion of pancreatic cancer cells through MUC5AC-mediated attenuation of E-cadherin. Oncogene 30: 714–723 20972463 10.1038/onc.2010.459

[bib25] Ingham PW, McMahon AP (2001) Hedgehog signaling in animal development: paradigms and principles. Genes Dev 15: 3059–3087 11731473 10.1101/gad.938601

[bib26] Jenkins D (2009) Hedgehog signalling: emerging evidence for non-canonical pathways. Cell Signal 21: 1023–1034 19399989 10.1016/j.cellsig.2009.01.033

[bib27] Kameda C, Tanaka H, Yamasaki A, Nakamura M, Koga K, Sato N, Kubo M, Kuroki S, Tanaka M, Katano M (2009) The Hedgehog pathway is a possible therapeutic target for patients with estrogen receptor-negative breast cancer. Anticancer Res 29: 871–880 19414322

[bib28] Kao J, Salari K, Bocanegra M, Choi YL, Girard L, Gandhi J, Kwei KA, Hernandez-Boussard T, Wang P, Gazdar AF, Minna JD, Pollack JR (2009) Molecular profiling of breast cancer cell lines defines relevant tumor models and provides a resource for cancer gene discovery. PLoS One 4: e6146 19582160 10.1371/journal.pone.0006146 PMC2702084

[bib29] Karhadkar SS, Bova GS, Abdallah N, Dhara S, Gardner D, Maitra A, Isaacs JT, Berman DM, Beachy PA (2004) Hedgehog signalling in prostate regeneration, neoplasia and metastasis. Nature 431: 707–712 15361885 10.1038/nature02962

[bib30] Kasper M, Regl G, Frischauf AM, Aberger F (2006) GLI transcription factors: mediators of oncogenic Hedgehog signalling. Eur J Cancer 42: 437–445 16406505 10.1016/j.ejca.2005.08.039

[bib31] Katoh Y, Katoh M (2009) Hedgehog target genes: mechanisms of carcinogenesis induced by aberrant hedgehog signaling activation. Curr Mol Med 9: 873–886 19860666 10.2174/156652409789105570

[bib32] Kinzler KW, Ruppert JM, Bigner SH, Vogelstein B (1988) The GLI gene is a member of the Kruppel family of zinc finger proteins. Nature 332: 371–374 2832761 10.1038/332371a0

[bib33] Kleer CG, van Golen KL, Braun T, Merajver SD (2001) Persistent E-cadherin expression in inflammatory breast cancer. Mod Pathol 14: 458–464 11353057 10.1038/modpathol.3880334

[bib34] Kubo M, Nakamura M, Tasaki A, Yamanaka N, Nakashima H, Nomura M, Kuroki S, Katano M (2004) Hedgehog signaling pathway is a new therapeutic target for patients with breast cancer. Cancer Res 64: 6071–6074 15342389 10.1158/0008-5472.CAN-04-0416

[bib35] Lauth M, Bergstrom A, Shimokawa T, Toftgard R (2007) Inhibition of GLI-mediated transcription and tumor cell growth by small-molecule antagonists. Proc Natl Acad Sci USA 104: 8455–8460 17494766 10.1073/pnas.0609699104 PMC1866313

[bib36] Lauth M, Toftgard R (2007) Non-canonical activation of GLI transcription factors: implications for targeted anti-cancer therapy. Cell Cycle 6: 2458–2463 17726373 10.4161/cc.6.20.4808

[bib37] Lee WY, Su WC, Lin PW, Guo HR, Chang TW, Chen HH (2004) Expression of S100A4 and Met: potential predictors for metastasis and survival in early-stage breast cancer. Oncology 66: 429–438 15452371 10.1159/000079496

[bib38] Li X, Deng W, Lobo-Ruppert S, Ruppert J (2007) Gli1 acts through Snail and E-cadherin to promote nuclear signaling by beta-catenin. Oncogene 26: 4489–4498 17297467 10.1038/sj.onc.1210241 PMC2233601

[bib39] Li X, Deng W, Nail C, Bailey S, Kraus M, Ruppert J, Lobo-Ruppert S (2006) Snail induction is an early response to Gli1 that determines the efficiency of epithelial transformation. Oncogene 25: 609–621 16158046 10.1038/sj.onc.1209077 PMC1361531

[bib40] Liang C, Park A, Guan J (2007) *In vitro* scratch assay: a convenient and inexpensive method for analysis of cell migration *in vitro*. Nat Protoc 2: 329–333 17406593 10.1038/nprot.2007.30

[bib41] Lo H, Zhu H, Cao X, Aldrich A, Ali-Osman F (2009) A novel splice variant of GLI1 that promotes glioblastoma cell migration and invasion. Cancer Res 69: 6790–6798 19706761 10.1158/0008-5472.CAN-09-0886 PMC2771365

[bib42] Mahindroo N, Connelly M, Punchihewa C, Kimura H, Smeltzer M, Wu S, Fujii N (2009) Structure activity relationships and cancer-cell selective toxicity of novel inhibitors of glioma-associated oncogene homologue 1 (Gli1) mediated transcription. J Med Chem 52: 361–372 10.1021/jm900106f PMC2853048 19545120

[bib43] Mori Y, Okumura T, Tsunoda S, Sakai Y, Shimada Y (2006) Gli-1 expression is associated with lymph node metastasis and tumor progression in esophageal squamous cell carcinoma. Oncology 70: 378–389 17179732 10.1159/000098111

[bib44] Mukherjee S, Frolova N, Sadlonova A, Novak Z, Steg A, Page GP, Welch DR, Lobo-Ruppert SM, Ruppert JM, Johnson MR, Frost AR (2006) Hedgehog signaling and response to cyclopamine differ in epithelial and stromal cells in benign breast and breast cancer. Cancer Biol Ther 5: 674–683 16855373 10.4161/cbt.5.6.2906 PMC1557635

[bib45] Nagai S, Nakamura M, Yanai K, Wada J, Akiyoshi T, Nakashima H, Ohuchida K, Sato N, Tanaka M, Katano M (2008) Gli1 contributes to the invasiveness of pancreatic cancer through matrix metalloproteinase 9 activation. Cancer Sci 99: 1377–1384 18410405 10.1111/j.1349-7006.2008.00822.x PMC11159230

[bib46] Neve RM, Chin K, Fridlyand J, Yeh J, Baehner FL, Fevr T, Clark L, Bayani N, Coppe JP, Tong F, Speed T, Spellman PT, DeVries S, Lapuk A, Wang NJ, Kuo WL, Stilwell JL, Pinkel D, Albertson DG, Waldman FM, McCormick F, Dickson RB, Johnson MD, Lippman M, Ethier S, Gazdar A, Gray JW (2006) A collection of breast cancer cell lines for the study of functionally distinct cancer subtypes. Cancer Cell 10: 515–527 17157791 10.1016/j.ccr.2006.10.008 PMC2730521

[bib47] Nieman M, Prudoff R, Johnson K, Wheelock M (1999) N-cadherin promotes motility in human breast cancer cells regardless of their E-cadherin expression. J Cell Biol 147: 631–644 10545506 10.1083/jcb.147.3.631 PMC2151177

[bib48] Nolan-Stevaux O, Lau J, Truitt ML, Chu GC, Hebrok M, Fernandez-Zapico ME, Hanahan D (2009) GLI1 is regulated through Smoothened-independent mechanisms in neoplastic pancreatic ducts and mediates PDAC cell survival and transformation. Genes Dev 23: 24–36 19136624 10.1101/gad.1753809 PMC2632164

[bib49] Pasca di Magliano M, Hebrok M (2003) Hedgehog signalling in cancer formation and maintenance. Nat Rev Cancer 3: 903–911 14737121 10.1038/nrc1229

[bib50] Pathi S, Pagan-Westphal S, Baker DP, Garber EA, Rayhorn P, Bumcrot D, Tabin CJ, Blake Pepinsky R, Williams KP (2001) Comparative biological responses to human Sonic, Indian, and Desert hedgehog. Mech Dev 106: 107–117 11472839 10.1016/s0925-4773(01)00427-0

[bib51] Pepinsky R, Zeng C, Wen D, Rayhorn P, Baker D, Williams K, Bixler S, Ambrose C, Garber E, Miatkowski K, Taylor FR, Wang EA, Galdes A (1998) Identification of a palmitic acid-modified form of human Sonic hedgehog. J Biol Chem 273: 14037–14045 9593755 10.1074/jbc.273.22.14037

[bib52] Quaranta V, Tyson DR, Garbett SP, Weidow B, Harris MP, Georgescu W (2009) Trait variability of cancer cells quantified by high-content automated microscopy of single cells. In Johnson ML, Brand L (eds) Methods Enzymol, pp 23–57. Academic Press: New York 10.1016/S0076-6879(09)67002-6 PMC2915824 19897088

[bib53] Riobo N, Manning D (2007) Pathways of signal transduction employed by vertebrate Hedgehogs. Biochem J 403: 369–370 17419683 10.1042/BJ20061723

[bib54] Riobo NA, Lu K, Ai X, Haines GM, Emerson Jr CP (2006a) Phosphoinositide 3-kinase and Akt are essential for Sonic Hedgehog signaling. Proc Natl Acad Sci USA 103: 4505–4510 16537363 10.1073/pnas.0504337103 PMC1450201

[bib55] Riobo NA, Lu K, Emerson Jr CP (2006b) Hedgehog signal transduction: signal integration and cross talk in development and cancer. Cell Cycle 5: 1612–1615 16880744 10.4161/cc.5.15.3130

[bib56] Rizvi S, DeMars C, Comba A, Gainullin V, Rizvi Z, Almada L, Wang K, Lomberk G, Fernández-Zapico M, Buttar N (2010) Combinatorial chemoprevention reveals a novel Smoothened-independent role of GLI1 in esophageal carcinogenesis. Cancer Res 70: 6787 20647328 10.1158/0008-5472.CAN-10-0197 PMC2954590

[bib57] Rouesse J, Friedman S, Sarrazin D, Mouriesse H, Le Chevalier T, Arriagada R, Spielmann M, Papacharalambous A, May-Levin F (1986) Primary chemotherapy in the treatment of inflammatory breast carcinoma: a study of 230 cases from the Institut Gustave-Roussy. J Clin Oncol 4: 1765–1771 3783202 10.1200/JCO.1986.4.12.1765

[bib58] Rubin LL, de Sauvage FJ (2006) Targeting the Hedgehog pathway in cancer. Nat Rev Drug Discov 5: 1026–1033 17139287 10.1038/nrd2086

[bib59] Ruiz A, Mas C, Stecca B (2007) The Gli code: an information nexus regulating cell fate, stemness and cancer. Trends Cell Biol 17: 438–447 17845852 10.1016/j.tcb.2007.06.007 PMC2601665

[bib60] Sanchez P, Hernandez AM, Stecca B, Kahler AJ, DeGueme AM, Barrett A, Beyna M, Datta MW, Datta S, Ruiz i Altaba A (2004) Inhibition of prostate cancer proliferation by interference with Sonic hedgehog-GLI1 signaling. Proc Natl Acad Sci USA 101: 12561–12566 15314219 10.1073/pnas.0404956101 PMC514658

[bib61] Scales S, de Sauvage F (2008) Mechanisms of Hedgehog pathway activation in cancer and implications for therapy. Trends Pharmacol Sci 30: 303–312 10.1016/j.tips.2009.03.007 19443052

[bib62] Singh B, Cook K, Martin C, Huang E, Mosalpuria K, Krishnamurthy S, Cristofanilli M, Lucci A (2010a) Evaluation of a CXCR4 antagonist in a xenograft mouse model of inflammatory breast cancer. Clin Exp Metastasis 27: 233–240 20229045 10.1007/s10585-010-9321-4

[bib63] Singh B, Irving L, Tai K, Lucci A (2010b) Overexpression of COX-2 in Celecoxib-resistant breast cancer cell lines. J Surg Res 163: 235–243 20691996 10.1016/j.jss.2010.04.061 PMC2943002

[bib64] Souzaki M, Kubo M, Kai M, Kameda C, Tanaka H, Taguchi T, Tanaka M, Onishi H, Katano M (2011) Hedgehog signaling pathway mediates the progression of non invasive breast cancer to invasive breast cancer. Cancer Sci 102: 373–381 21091847 10.1111/j.1349-7006.2010.01779.x PMC11159393

[bib65] Stecca B, Mas C, Clement V, Zbinden M, Correa R, Piguet V, Beermann F, Ruiz IAA (2007) Melanomas require Hedgehog-GLI signaling regulated by interactions between GLI1 and the RAS-MEK/AKT pathways. Proc Natl Acad Sci USA 104: 5895–5900 17392427 10.1073/pnas.0700776104 PMC1838820

[bib66] Stecca B, Ruiz I (2010) Context-dependent regulation of the GLI code in cancer by hedgehog and non-hedgehog signals. J Mol Cell Biol 2: 84–95 20083481 10.1093/jmcb/mjp052 PMC2905064

[bib67] Streicher K, Willmarth N, Garcia J, Boerner J, Dewey T, Ethier S (2007) Activation of a nuclear factor {kappa} B/interleukin-1 positive feedback loop by amphiregulin in human breast cancer cells. Mol Cancer Res 5: 847–861 17670913 10.1158/1541-7786.MCR-06-0427

[bib68] Taipale J, Chen JK, Cooper MK, Wang B, Mann RK, Milenkovic L, Scott MP, Beachy PA (2000) Effects of oncogenic mutations in Smoothened and Patched can be reversed by cyclopamine. Nature 406: 1005–1009 10984056 10.1038/35023008

[bib69] Teglund S, Toftgård R (2010) Hedgehog beyond medulloblastoma and basal cell carcinoma. Biochim Biophys Acta 1805: 181–208 20085802 10.1016/j.bbcan.2010.01.003

[bib70] Ten Haaf A, Bektas N, Von Serenyi S, Losen I, Arweiler E, Hartmann A, Knüchel R, Dahl E (2009) Expression of the glioma-associated oncogene homolog (GLI) 1 in human breast cancer is associated with unfavourable overall survival. BMC Cancer 9: 298 19706168 10.1186/1471-2407-9-298 PMC2753634

[bib71] Testaz S, Jarov A, Williams K, Ling L, Koteliansky V, Fournier-Thibault C, Duband J (2001) Sonic hedgehog restricts adhesion and migration of neural crest cells independently of the Patched-Smoothened-Gli signaling pathway. Proc Natl Acad Sci USA 98: 12521–12526 11592978 10.1073/pnas.221108698 PMC60086

[bib72] Theunissen J, de Sauvage F (2009) Paracrine Hedgehog signaling in cancer. Cancer Res 69: 6007–6010 19638582 10.1158/0008-5472.CAN-09-0756

[bib73] Van Laere S, Limame R, Van Marck E, Vermeulen P, Dirix L (2010) Is there a role for mammary stem cells in inflammatory breast carcinoma? Cancer 116: 2794–2805 20503411 10.1002/cncr.25180

[bib74] Van Laere S, Van der Auwera I, Van den Eynden G, Van Hummelen P, Van Dam P, Van Marck E, Vermeulen P, Dirix L (2007) Distinct molecular phenotype of inflammatory breast cancer compared to non-inflammatory breast cancer using Affymetrix-based genome-wide gene-expression analysis. Br J Cancer 97: 1165–1174 17848951 10.1038/sj.bjc.6603967 PMC2360452

[bib75] Wang K, Pan L, Che X, Cui D, Li C (2010a) Sonic Hedgehog/GLI1 signaling pathway inhibition restricts cell migration and invasion in human gliomas. Neurol Res 32: 975–980 20444323 10.1179/016164110X12681290831360

[bib76] Wang L, Liu Z, Gambardella L, Delacour A, Shapiro R, Yang J, Sizing I, Rayhorn P, Garber E, Benjamin C, Williams KP, Taylor FR, Barrandon Y, Ling L, Burkly LC (2000) Conditional disruption of hedgehog signaling pathway defines its critical role in hair development and regeneration. J Investig Dermatol 114: 901–908 10771469 10.1046/j.1523-1747.2000.00951.x

[bib77] Wang X, Osada T, Wang Y, Yu L, Sakakura K, Katayama A, McCarthy J, Brufsky A, Chivukula M, Khoury T, Hsu DS, Barry WT, Lyerly HK, Clay TM, Ferrone S (2010b) CSPG4 protein as a new target for the antibody-based immunotherapy of triple-negative breast cancer. J Natl Cancer Inst 102: 1496–1512 20852124 10.1093/jnci/djq343 PMC2950168

[bib78] Williams JA, Guicherit OM, Zaharian BI, Xu Y, Chai L, Wichterle H, Kon C, Gatchalian C, Porter JA, Rubin LL, Wang FY (2003) Identification of a small molecule inhibitor of the hedgehog signaling pathway: effects on basal cell carcinoma-like lesions. Proc Natl Acad Sci USA 100: 4616–4621 12679522 10.1073/pnas.0732813100 PMC153604

[bib79] Williams KP, Rayhorn P, Chi-Rosso G, Garber EA, Strauch KL, Horan GS, Reilly JO, Baker DP, Taylor FR, Koteliansky V, Pepinsky RB (1999) Functional antagonists of sonic hedgehog reveal the importance of the N terminus for activity. J Cell Sci 112: 4405–4414 10564658 10.1242/jcs.112.23.4405

[bib80] Willmarth N, Ethier S (2006) Autocrine and juxtacrine effects of amphiregulin on the proliferative, invasive, and migratory properties of normal and neoplastic human mammary epithelial cells. J Biol Chem 281: 37728–37737 17035230 10.1074/jbc.M606532200

[bib81] Wu M, Wu Z, Rosenthal D, Rhee E, Merajver S (2010) Characterization of the roles of RHOC and RHOA GTPases in invasion, motility, and matrix adhesion in inflammatory and aggressive breast cancers. Cancer 116: 2768–2782 20503409 10.1002/cncr.25181

[bib82] Xu L, Kwon Y, Frolova N, Steg A, Yuan K, Johnson M, Grizzle W, Desmond R, Frost A (2010) Gli1 promotes cell survival and is predictive of a poor outcome in ER-negative breast cancer. Breast Cancer Res Treat 123: 59–71 10.1007/s10549-009-0617-5 PMC2888711 19902354

[bib83] Yamauchi H, Cristofanilli M, Nakamura S, Hortobagyi G, Ueno N (2009) Molecular targets for treatment of inflammatory breast cancer. Nature Rev Clin Oncol 6: 387–394 19468291 10.1038/nrclinonc.2009.73

[bib84] Yang L, Xie G, Fan Q, Xie J (2009) Activation of the hedgehog-signaling pathway in human cancer and the clinical implications. Oncogene 29: 469–481 19935712 10.1038/onc.2009.392

[bib85] Yauch RL, Gould SE, Scales SJ, Tang T, Tian H, Ahn CP, Marshall D, Fu L, Januario T, Kallop D, Nannini-Pepe M, Kotkow K, Marsters JC, Rubin LL, de Sauvage FJ (2008) A paracrine requirement for hedgehog signalling in cancer. Nature 455: 406–410 18754008 10.1038/nature07275

[bib86] Zhang X, Harrington N, Moraes RC, Wu MF, Hilsenbeck SG, Lewis MT (2008) Cyclopamine inhibition of human breast cancer cell growth independent of Smoothened (Smo). Breast Cancer Res Treat 115: 505–521 18563554 10.1007/s10549-008-0093-3 PMC5300001

[bib87] Zhao J, Chen G, Cao D, Li Y, Diao F, Cai H, Jin Y, Lu J (2009) Expression of Gli1 correlates with the transition of breast cancer cells to estrogen-independent growth. Breast Cancer Res Treat 119: 39–51 19191023 10.1007/s10549-009-0323-3

