# Supplementary figures and images for: Targeting GLI1 expression in human inflammatory breast cancer cells enhances apoptosis and attenuates migration
Source: Br J Cancer. 2011 Apr 19;104(10):1575–86. doi: 10.1038/bjc.2011.133 (PMC3101910; doi:10.1038/bjc.2011.133)

## Slide 1
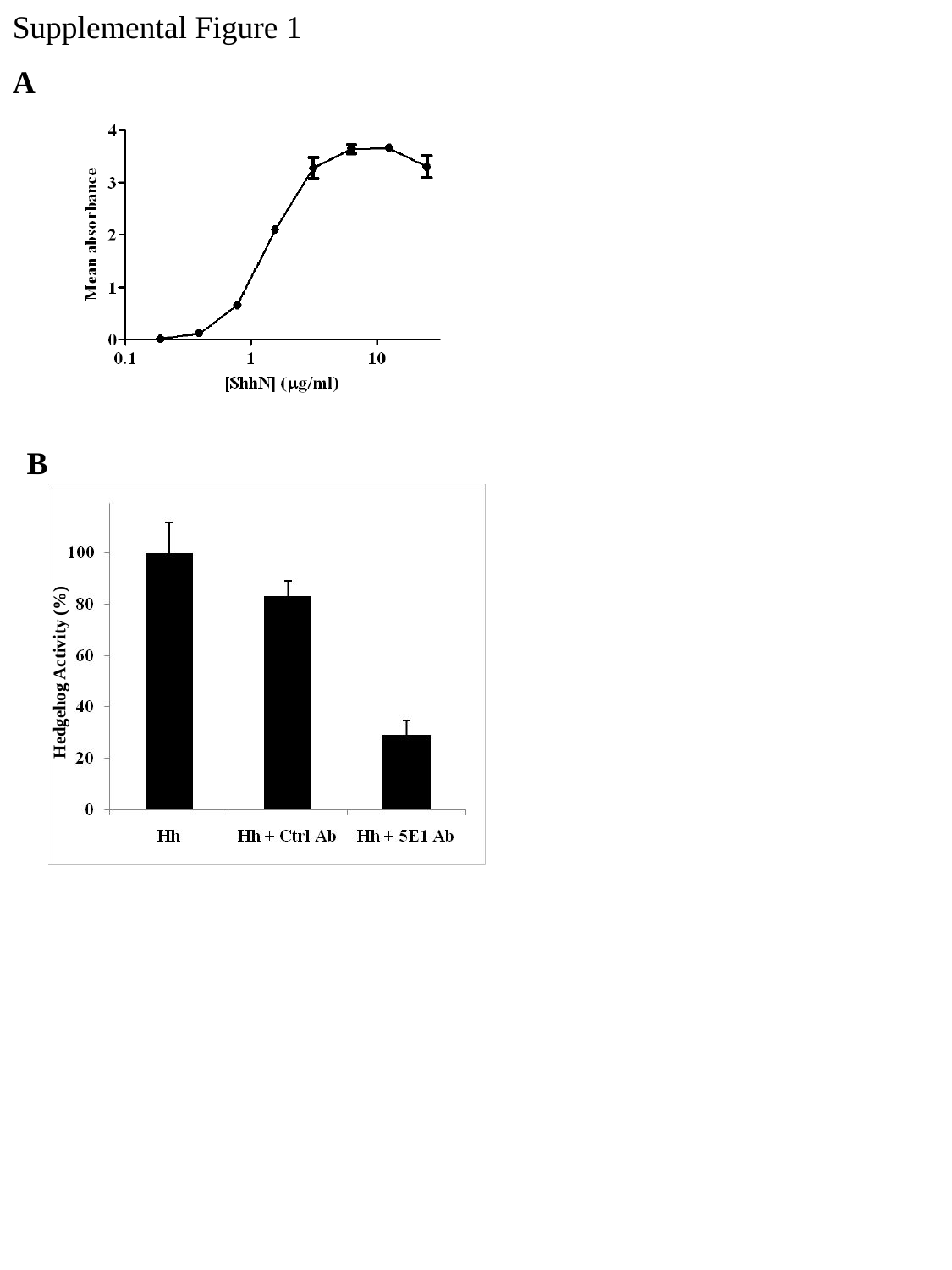

Supplemental Figure 1
A
B
Hedgehog Activity (%)

Supplement: Supplementary Figure 1 [file bjc2011133x1.ppt]
