# Supplementary material for: Targeting GLI1 expression in human inflammatory breast cancer cells enhances apoptosis and attenuates migration
Source: Br J Cancer. 2011 Apr 19;104(10):1575–86. doi: 10.1038/bjc.2011.133 (PMC3101910; doi:10.1038/bjc.2011.133)
Supplement: Supplementary Figure Legends [file bjc2011133x2.doc]

**Supplemental Figure 1** Activity of ShhN protein and effect of the anti-Hh 5E1 antibody on ShhN activity in C3H10T1/2 cells. (**A**) The activity of recombinant ShhN was assessed in the C3H10T1/2 alkaline phosphatase (AP) induction assay. Serial 2-fold dilutions of ShhN were incubated with the cells for 4 days and the resulting levels of AP activity measured at 405 nm using the AP substrate para–nitrophenyl phosphate. (**B**) Anti-Hh 5E1 Ab and control Ab were incubated with C3H10T1/2 cells and ShhN protein (2 µg/ml) for 4 days and activity measured as in A. Results presented as percent activity relative to ShhN protein alone. All measurements carried out in triplicate.
